# Hourly Engagement Patterns of a Wearable Monitor Unveil Key Attention Windows for Hypertension Management Strategies

**DOI:** 10.1016/j.jacadv.2024.101384

**Published:** 2025-01-08

**Authors:** Tiago P. Almeida, David Perruchoud, Joachim Zahnd, Josep Sola, Jay Shah, Gregoire Wuerzner

**Affiliations:** a Core Technology, Aktiia SA, Neuchâtel, Switzerland; b Lausanne University Hospital and University of Lausanne, Lausanne, Switzerland

**Figure:**
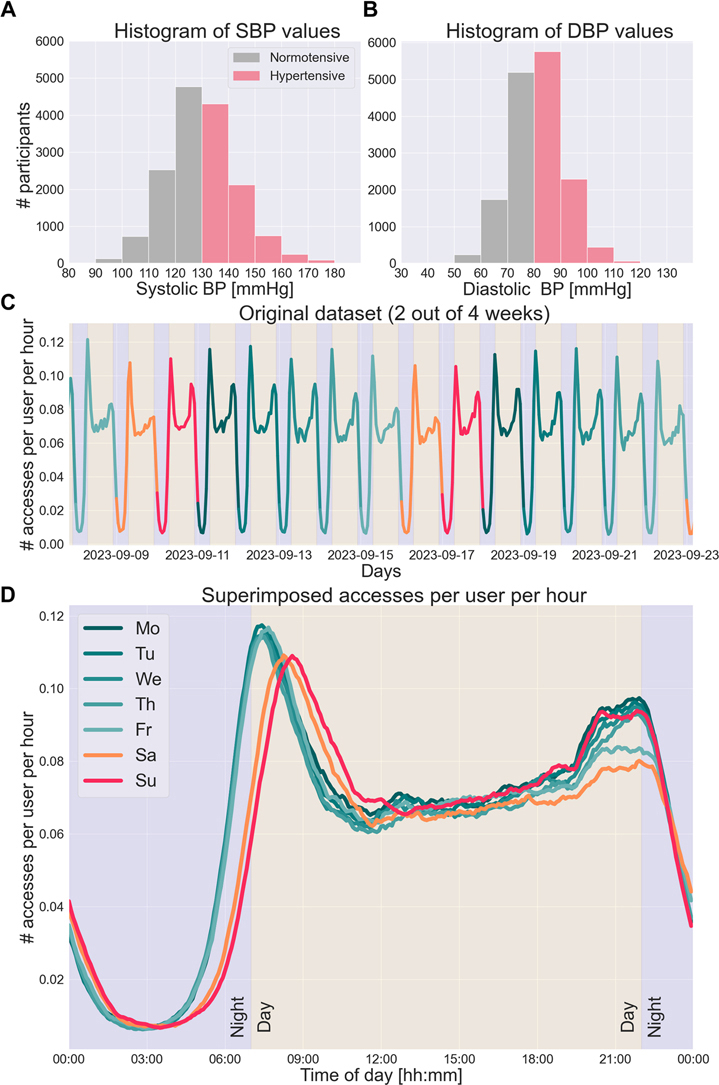

Wearable digital health technologies have gained in popularity across various health care applications. Research indicates a surge in wearable usage, with approximately 71% annual growth in 2019 and a similar trend estimated toward 2023.^1^ Additionally, a comprehensive review has confirmed that wearable technology boosts patient engagement and adherence to treatment regimes, ultimately improving health outcomes.^2^

Recent technological advancements have made it possible to measure blood pressure (BP) via photoplethysmographic signals, eliminating the routine need for the cuff.^3^ This innovation paves the way for a new era in BP monitoring with wearable, cuffless devices. Accordingly, 92% of smartwatch owners use such data to maintain and manage their health, which now can include BP monitoring.^4^ Studies confirm that self-monitoring with home BP monitors helps to better control BP.^5^ However, the use of home BP monitors at the recommended frequency is limited.^6^ Cuffless BP monitors, therefore, emerge as the promising solution for continual, long-term BP monitoring.

Recent studies have explored general population engagement with wearable devices, focusing on broader trends rather than providing detailed hourly analysis.^4^^,^^7^ Despite their important contributions, these studies lack the granularity needed for hour-by-hour behavioral insights. In the present letter, we provide first-hand hour-by-hour interaction of the general population with a wearable for BP monitoring without any guidance or recommendation.

Data from 15,740 European residents (age 57.9 ± 11.1 years, 21.8% female, body mass index 27.9 ± 4.9 kg/m^2^) were included in the investigation. All users voluntarily purchased and wore a validated, Conformité Européene-marked, over-the-counter cuffless wrist BP monitor (Aktiia SA).^3^ All users provided permission to use their anonymous and/or aggregated data for research purposes through the commercial agreement of usage for the Aktiia monitor. The Aktiia monitor is comprised of a bracelet that collects green reflective photoplethysmographic signals from the wrist, an oscillometric brachial cuff used for initializations (ie calibrations) performed at least once a month,^3^ and a smartphone application. Every time the user accesses the smartphone application—ie, synchronization—the data from the bracelet are transferred via Bluetooth and forwarded to Aktiia’s cloud server, where they are stored. The smartphone application also displays the BP values measured by the Aktiia monitor and can be checked at any time by the user.

**Figure 1:**
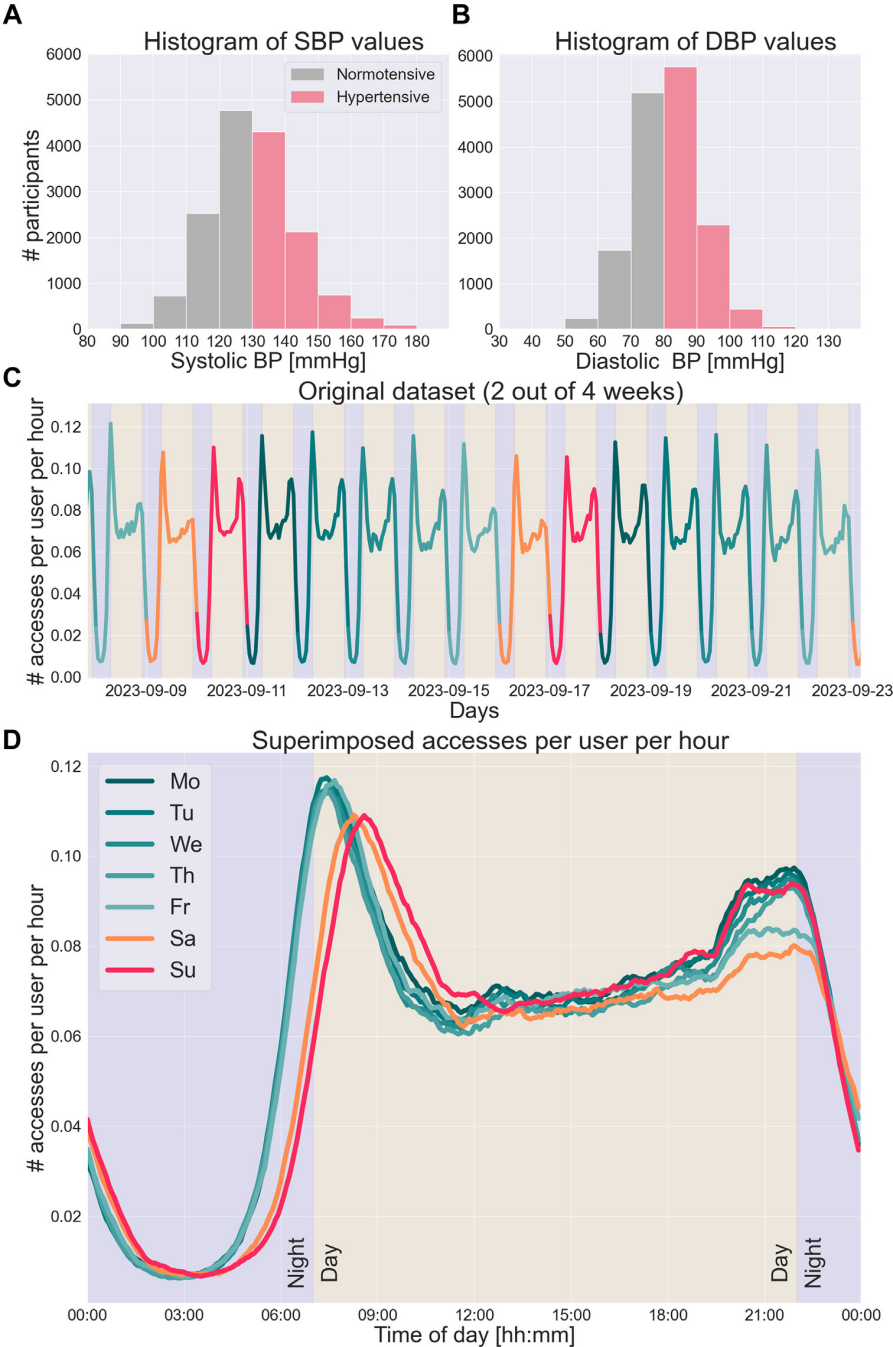
Distribution of BP Measurements and User Engagement Patterns in the Study Cohort (A) Histogram of SBP values, with a single measurement per participant, collected using the oscillometric cuff in the study cohort (hypertension SBP ≥130 mm Hg). (B) Histogram of DBP values, with a single measurement per participant, collected using the oscillometric cuff in the study cohort (hypertension DBP ≥80 mm Hg). (C) The number of accesses to the Aktiia monitor’s smartphone application performed by hour for 2 weeks (out of 4 included in the study) color-coded by the day of the week. (D) The number of accesses performed per hour per user, superimposed by day of the week, considering the 4 weeks included in the study. DBP = diastolic blood pressure; SBP = systolic blood pressure.

Based on data collected from the oscillometric cuff, 9,982 of the 15,740 monitored individuals (63.4%) were classified as hypertensive according to the U.S. guidelines (systolic BP ≥130 mm Hg or diastolic BP ≥80 mm Hg).^8^ Figures 1A and 1B illustrate the distributions of systolic BP and diastolic BP values collected using the oscillometric cuff (a single measurement per participant). The number of hourly app accesses for 4 weeks (01-28 September 2023) were retrospectively analyzed (641,896 total synchronizations, median: 24 [interquartile range 9-52] synchronizations per user). The hourly count was established using a 1-hour-centered rolling window with a 5-minute step. The number of hourly synchronizations performed for 2 of the 4 weeks is shown in Figure 1C. The result shows clear patterns of behavior of the general population with specific attention windows, one early in the morning and another before bedtime. Figure 1D illustrates the number of synchronizations performed by hour per user, superimposed by day of the week, highlighting changes in the attention windows according to each day of the week. A clear weekly pattern of interest was noticed from Sunday evening until Friday morning, with reduced interest in self-monitoring between Friday evening until Sunday morning. A noticeable lag of approximately 1 hour on weekend mornings suggests users wake up later during weekends. Results from low-engagement users (3,950 users in the least-engaged quartile, with 1-9 accesses) showed similar behavioral patterns to the general population (not shown here).

This is the first large-scale population-based study to provide insights into how users interact with wearable BP monitors hour-by-hour throughout the day. The results show distinct hourly and weekly patterns of interest that indicate specific attention windows when users typically check their BP values. These patterns suggest the emergence of habitual use, pointing to a potential “hooking” effect with wearable BP monitors.^9^ While the motivations for uploading data remain unknown, they may include checking day/night-time BP variability, checking BP in case of symptoms, generating a report for family members or physicians, long-term monitoring of BP, and/or monitoring of changes based on lifestyle changes. Conversely, a recent study highlighted the risk of potential addiction to mobile applications, emphasizing the need for guidelines and policy interventions to mitigate challenges in mobile applications addiction.^10^ However, a responsible and conscientious approach toward the data provided by wearables to promote healthy behaviors—eg, the hourly patterns of engagement—could potentially transform health care delivery. Understanding the attention windows when users check their BP enables the development of personalized strategies. For example, gamification could encourage users to engage in activities such as moving, exercising, or taking breaks, leading to improved overall health outcomes and BP reduction. Hourly engagement patterns could also help identify periods when users are likely to engage in behaviors that raise BP, such as consuming high-sodium foods or experiencing stress. For instance, if data suggest an increase in BP for several days, this could be correlated to a decrease in physical activity, a decrease in antihypertensive drug adherence, or a change in diet—all of which can be monitored on smartphone possibly simultaneously in the near future. Monitoring behavior is often the first step in a change of attitude and would reinforce patient empowerment. Furthermore, understanding when users check their BP can aid in developing personalized lifestyle interventions. If a user checks their BP after a workout, the system could provide positive reinforcement, such as congratulatory messages or tips on maintaining the workout routine. Conversely, if a user’s BP readings remain high despite regular checks, the system might suggest increasing physical activity, making dietary adjustments, or consulting a health care provider. Additionally, notifications based on personalized attention windows can be sent to encourage adherence to prescribed antihypertensive medications in users with uncontrolled BP. These strategies could positively influence user habits, paving the way for the creation of targeted, data-driven interventions that can substantially improve hypertension management.

While it would be interesting to explore potential correlations between BP and symptoms or medication titration, the present study utilized data from real-world users collected as they carried out their normal routines. Consequently, the users were not asked to disclose potential symptoms or medication use during system setup. Nevertheless, we believe the results presented here are representative of population behavior, and the patterns of engagement identified could be translated into actionable strategies for personalized health care delivery.

**What is the clinical question being addressed?** The hour-by-hour interaction of the general population with health care wearables could support the creation of innovative data-driven interventions. Therefore, our research question was: Are there hourly patterns of engagement with a blood pressure wearable that could be used for tailored intervention?

**What is the main finding?** Our data show distinct hourly patterns of interest that indicate specific attention windows (mornings and evenings, especially during weekdays) when users interact with wearables. These patterns suggest the emergence of habitual use that could be used to tailor personalized health care strategies.
